# Brain’s Reward Circuits Mediate Itch Relief. A Functional MRI Study of Active Scratching

**DOI:** 10.1371/journal.pone.0082389

**Published:** 2013-12-06

**Authors:** Alexandru D. P. Papoiu, Leigh A. Nattkemper, Kristen M. Sanders, Robert A. Kraft, Yiong-Huak Chan, Robert C. Coghill, Gil Yosipovitch

**Affiliations:** 1 Department of Dermatology, Wake Forest School of Medicine, Winston-Salem, North Carolina, United States of America; 2 Department of Neurobiology & Anatomy, Wake Forest School of Medicine, Winston-Salem, North Carolina, United States of America; 3 Department of Regenerative Medicine, Wake Forest School of Medicine, Winston-Salem, North Carolina, United States of America; 4 Department of Biomedical Engineering, Wake Forest University & Virginia Tech, Winston-Salem, North Carolina, United States of America; 5 PhD Program in Neurosciences, Graduate School of Biomedical Sciences, Wake Forest University, Winston-Salem, North Carolina, United States of America; 6 Biostatistics Unit, Yong Loo Lin School of Medicine, National University of Singapore, Singapore, Singapore; University of California, San Francisco, United States of America

## Abstract

Previous brain imaging studies investigating the brain processing of scratching used an exogenous intervention mimicking scratching, performed not by the subjects themselves, but delivered by an investigator. In real life, scratching is a conscious, voluntary, controlled motor response to itching, which is directed to the perceived site of distress. In this study we aimed to visualize in real-time by brain imaging the core mechanisms of the itch-scratch cycle when scratching was performed by subjects themselves. Secondly, we aimed to assess the correlations between brain patterns of activation and psychophysical ratings of itch relief or pleasurability of scratching. We also compared the patterns of brain activity evoked by self-scratching vs. passive scratching. We used a robust tridimensional Arterial Spin Labeling fMRI technique that is less sensitive to motion artifacts: 3D gradient echo and spin echo (GRASE) - Propeller. Active scratching was accompanied by a higher pleasurability and induced a more pronounced deactivation of the anterior cingulate cortex and insula, in comparison with passive scratching. A significant involvement of the reward system including the ventral tegmentum of the midbrain, coupled with a mechanism deactivating the periaqueductal gray matter (PAG), suggests that itch modulation operates in reverse to the mechanism known to suppress pain. Our findings not only confirm a role for the central networks processing reward in the pleasurable aspects of scratching, but also suggest they play a role in mediating itch relief.

## Introduction

Scratching is the natural response to itch and, by definition, inseparable from it. The act of scratching not only diminishes itch, but can be rewarding and addictive. The itch-scratch cycle is a complex phenomenon involving sensory, motor and emotional components. It is well-known that the urge to scratch can be remarkably intense, since the reward provided by scratching brings itch relief and associated feelings of pleasurability. Recent studies have shown that rating scratching as a pleasurable experience is correlated with the intensity of the underlying itch, both in patients with chronic itch [1] and healthy individuals [2].

Previous brain imaging studies have employed BOLD fMRI to examine the effects evoked by scratching. However, these studies used “passive scratching” performed by an investigator using a device, such as a cytology brush [3] or a copper wire [4]. In real life, scratching is a conscious, controlled motor response, targeted to the perceived site of itch, and continuously adjusted by sensory feedback in a manner that seeks to provide relief. Therefore, in the current study, we used fMRI to visualize the stages of the itch-scratch cycle, when scratching was performed by study subjects themselves. We also aimed to investigate whether active and passive scratching had differential effects on the pleasurable aspects of scratching, on the degree of itch relief, and the manner in which they were uniquely correlated with specific itch-relief patterns of brain activation or deactivation. 

We opted to include both modalities of scratching in our study in order to dissect the characteristic features of the itch-scratch cycle, when scratching was controlled and performed by subjects themselves in response to the itch they perceived. In this regard, passive scratching provided a control condition, offering only the mechanical stimulation that reduces itch, but missing the intricate, inner feedback loops which would close within the same person’s brain, when placed at the center and in control of the action, instead at the receiving end. We therefore specifically aimed to contrast these two conditions, to better illuminate the intimate rapport between sensory perception and motor action, and to observe how this interplay would be reflected in the mediation of itch relief. It is known in the field of reward that there are different operational modes: one which is dependent on one’s own actions (a contingent form of reward) and one that is exogenously provided and passively received. Thus, one of our aims was to uncover potentially meaningful differences between these two forms of relief. An additional rationale to include both forms of scratching in the present study was that previous research papers on this topic have used a passive form of scratching. Therefore, we intended to see how our results would compare to the previous reports and decided to study both active and passive forms of scratching, within the confines and internal controls of the same study, using the same methodology.

## Results

### Psychophysical data

**Figure 1:**
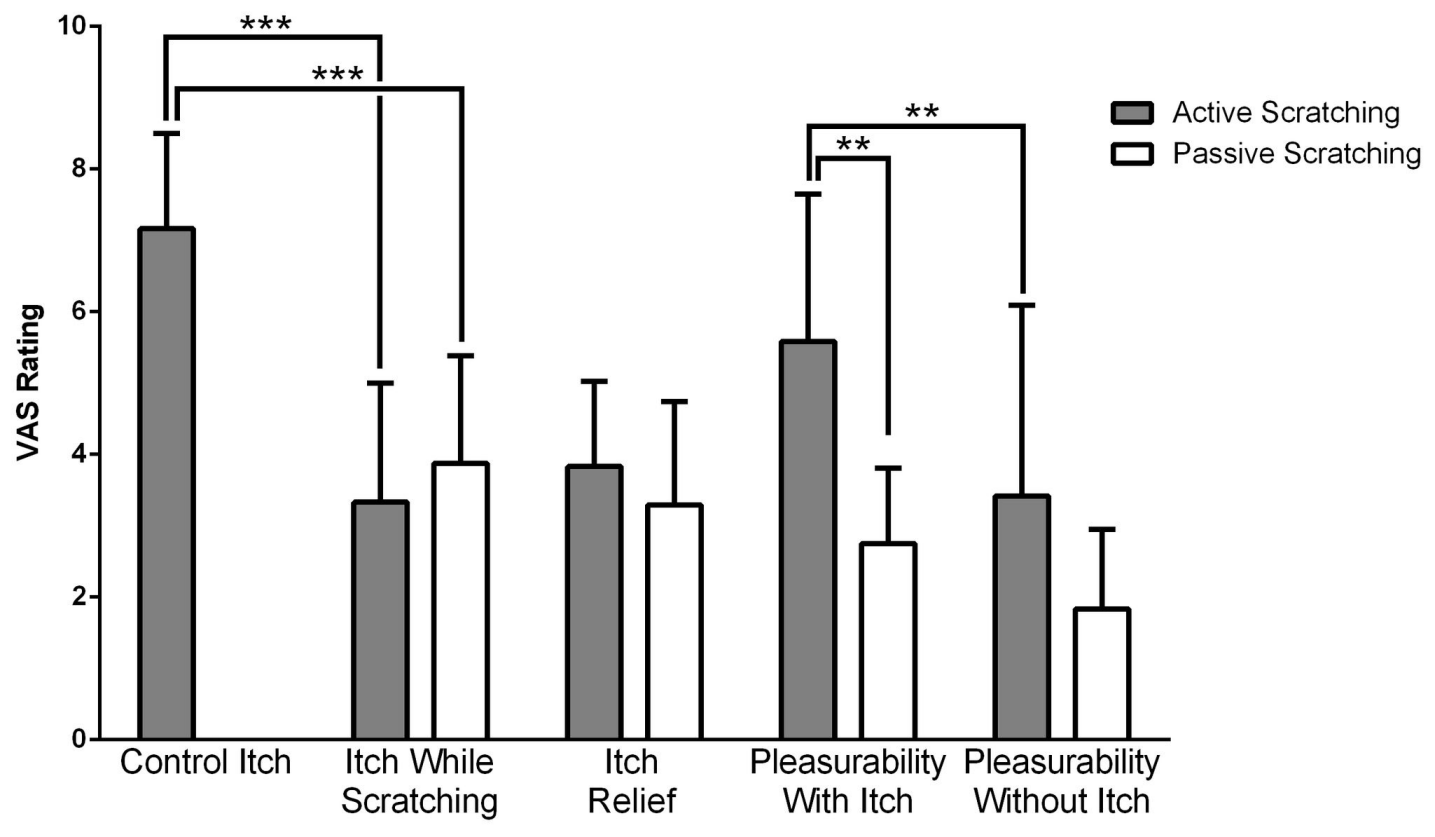
Visual analog scale ratings of itch intensity, itch relief and of the associated pleasurability during active scratching performed by subjects themselves, or passive scratching performed by an investigator. Ratings were recorded immediately at the end of each fMRI scan. (*** p<0.001; ** p<0.01).

All subjects responded to itch stimulation, with an average itch intensity of 7.2±1.3 (on a scale of 0-10). Visual Analog Scale (VAS) ratings of the perceived itch intensity, of the pleasurability of scratching and the magnitude of itch relief provided by active scratching or passive scratching are presented in Figure 1. Active scratching decreased itch intensity from 7.2 to 3.31 (p<0.0001), while passive scratching decreased itch to 3.90 (p <0.0001). In other words, itch relief induced by active scratching was 3.8±1.2, while the relief afforded by passive scratching was 3.3±1.5. Active scratching was associated with a significantly higher pleasurability (p<0.01) compared to passive scratching. Both active and passive scratching were rated more pleasurable in the presence of itch (5.6 ± 2.1 active; 3.3 ± 1.1 passive) than in its absence (3.5±2.6 AS; 1.8 ±1.1 PS, p<0.05). Active scratching was significantly more pleasurable than passive scratching, not only in the presence of itch, but also in the absence of itch (p<0.01 and p<0.05, respectively). 

###  The brain processing of active scratching an itch enables the visualization of the itch-scratch cycle

**Figure 2:**
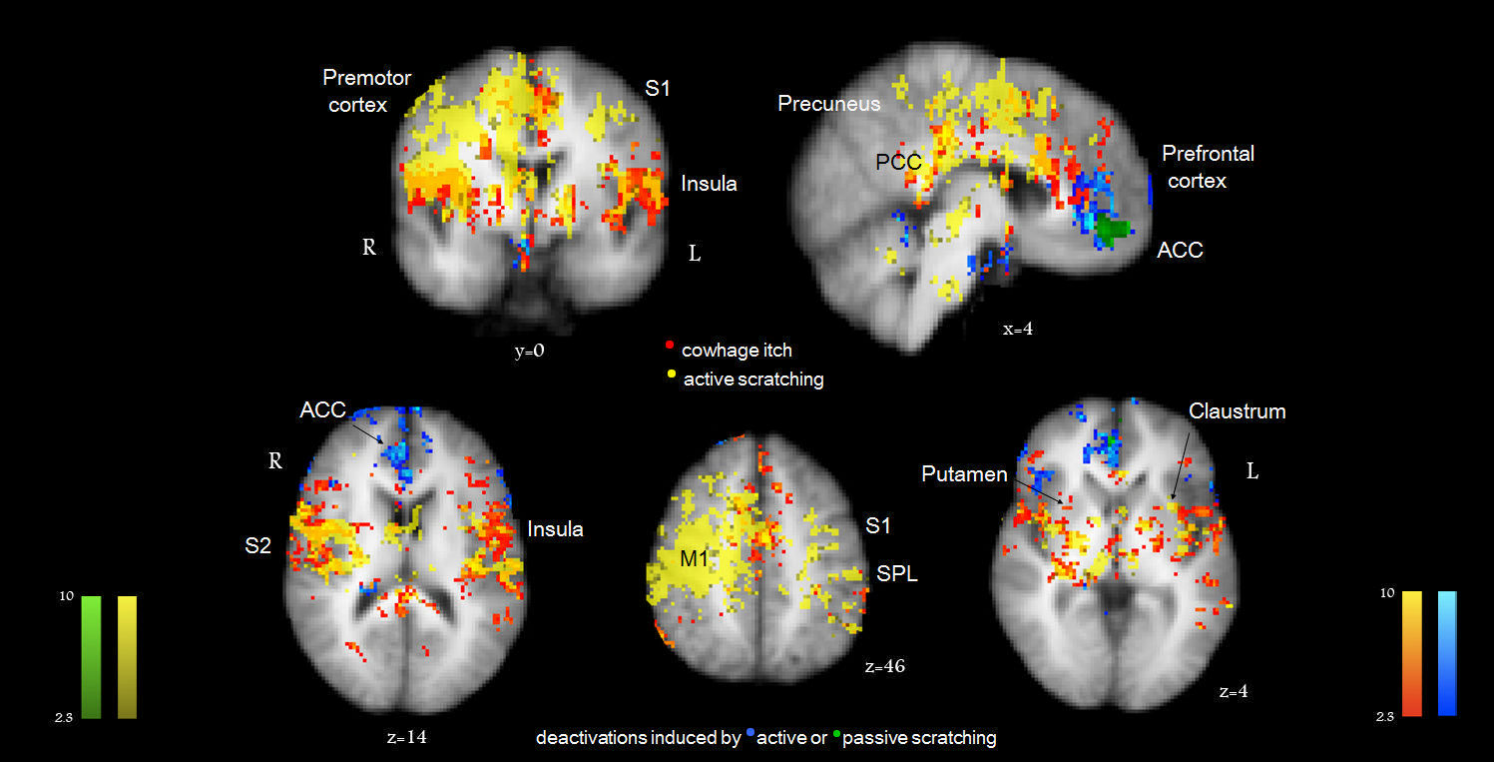
Overview of the itch-scratch cycle. Activations induced by actively scratching an itch (yellow), main areas activated by itch (red-orange), and major areas of deactivations induced by both forms of scratching are shown (with active scratching in blue, passive scratching in green). Standard Talairach space coordinates (x, y, z). The color tones displayed correspond to Z score values as depicted in the color bars (at the same scale). R = Right; L = Left.

**Figure 3:**
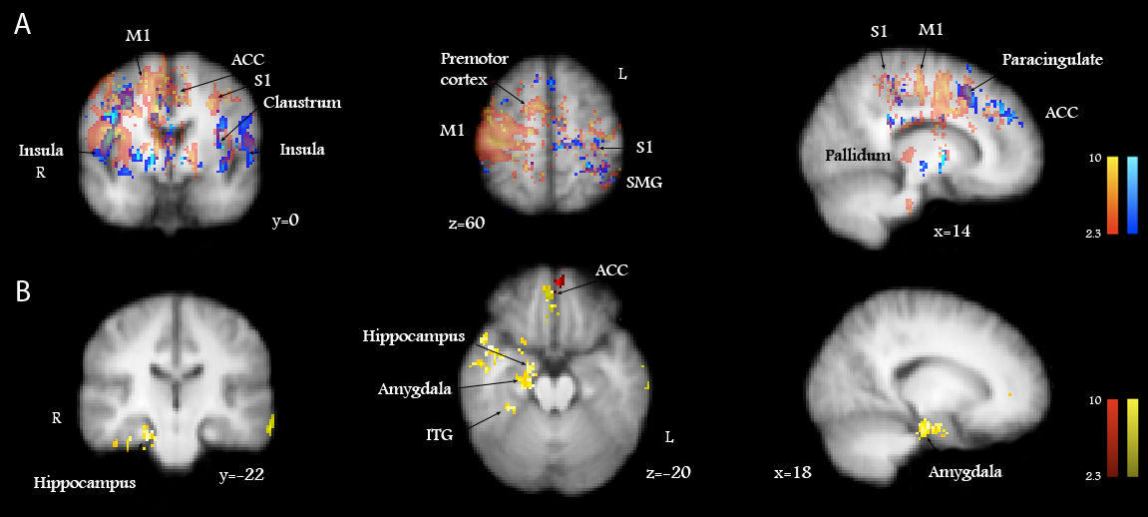
A) Brain activations induced by active scratching an itch (red) are displayed together with the activations induced by the passive scratching an itch (blue), analyzed as stand-alone conditions. B) Deactivations induced by active scratching (yellow) were represented in the hippocampus, amygdala, ACC and only slightly overlapped with the deactivations induced by passive scratching in the ACC (red). Standard Talairach space coordinates (x, y, z). The color tones displayed correspond to Z score values as depicted in the color bars (at the same scale). R = Right; L = Left.

In this section, we describe the complex patterns of activation observed during the continuous brain processing of actively scratching an itch (presented in Figure 2). Our study design - having the left hand scratching the right forearm which experienced itch - enabled the observation of a relatively segregated processing of motor and sensory information within the two cerebral hemispheres. The motor activity was predominantly localized to the right primary motor (M1) and premotor areas, with associated activations of the corticospinal tract, midbrain structures, basal ganglia, cerebellum and supplementary motor areas (SMA) of the right hemisphere (Figure 2). The sensory input related to itch was primarily relayed through the thalamus (ventral posterolateral nucleus, VPL) and was observed to activate the S1 of the left hemisphere and S2 bilaterally (Figures 2-3). Much less extensive ipsilateral activations were observed for M1 and S1 areas (i.e. ipsilateral to the itch site). The sensory input from the mechanical action of scratching alone (in the absence of itch) evoked activations of left S1 in an area which was adjacent to, but did not overlap with the areas activated by itch. 

**Figure 4:**
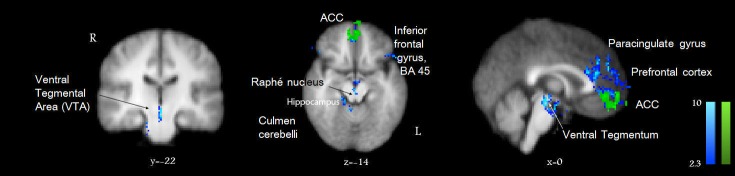
The dynamic effect of active or passive scratching an itch were analyzed in direct contrast to the itch condition. Actively scratching an itch (blue) deactivated the ACC, the ventromedial prefrontal Cortex, midbrain (VTA), while the effect of passive scratching was found only in the ACC (green). Standard Talairach space coordinates (x, y, z). The color tones displayed correspond to Z score values as depicted in the color bars (at the same scale). R = Right; L = Left.

The deactivations evoked by active scratching an itch were significantly represented in the following areas: ACC (Right, R), amygdala (R), cerebellum (anterior lobe, culmen; R), frontal medial cortex (R), frontal pole (bilateral), hippocampus (R), inferior frontal gyrus (Left, L), insula (R), and paracingulate gyrus (bilateral) (Figure 4A). In contrast, passive scratching of an itch significantly deactivated only four major areas: ACC (mostly L), frontal medial cortex, frontal pole, and paracingulate gyrus (bilateral), while scratching alone in the absence of itch did not evoke significant deactivations. The higher-level contrast analysis between active and passive scratching an itch revealed that the ACC (BA25, 24, 32), the frontal medial cortex (BA10) and frontal pole (BA11) were more extensively activated by active scratching in comparison with passive scratching. The amygdala, hippocampus and the parahippocampal gyrus (ipsilateral to itch) were significantly deactivated only by active scratching (Figures 3B, 4). 

At the level of **midbrain**, active scratching activated not only formations involved in motor coordination (red nucleus, substantia nigra), but also the ventral tegmental area (VTA) and a midline structure consistent with the location of raphé nucleus, slightly extending into nucleus cuneiformis. These activations also extended into the upper pons, implicating the rostromedial tegmentum (the tail of the VTA). Therefore, we observed that important reward circuit stations in the midbrain were significantly recruited by active scratching. Notably, passive scratching did not involve midbrain structures to a large extent, but activated the subthalamic nucleus, related to reward processing [5]. The periaqueductal gray (PAG) was significantly deactivated by active scratching with a high Z score (>6), and this effect was significantly correlated with the perceived pleasurability. 

In order to gain further insight into the intricate mechanism of self-scratching, we have performed the following higher-level contrast analyses. We analyzed brain activation patterns in respect to activations evoked by itch itself and compared active with passive scratching in the presence or absence of itch. The areas that were significantly deactivated by scratching an itch either passively or actively are shown in Figure 3. The contrast between the patterns of actively scratching an itch and itch alone revealed which itch-related structures were significantly impacted, being deactivated by scratching (Figure 4). The most important deactivations evoked by active scratching of itch were represented in the ACC, paracingulate gyrus, frontal pole and medial frontal cortex, mammillary bodies, hippocampus and parahippocampal gyrus, as well as in the discrete regions of the midbrain: the VTA and raphé nucleus. These results suggest that stations previously described to be implicated in reward circuits are recruited in the brain processing of self-scratching an itch. Therefore, it is suggested that these regions underlie the associated pleasurability and the addictive features of scratching.

In order to identify the specific features of scratching an itch in comparison with scratching alone (in the absence of itch), we performed a higher level contrast analysis between these two conditions. Interestingly, the primary motor cortex, the premotor cortex and somatosensory area S1 were more strongly activated by scratching in the presence of itch compared to scratching without an itch. Areas related to emotion (ACC), craving (nucleus accumbens, NAc) and motivation (putamen) were emphasized in the context of scratching an itch. An overview summarizing the effects induced by the two forms of scratching in various cerebral regions of interest is presented in Table S1.

### Brain areas associated with the pleasurability of scratching

**Figure 5:**
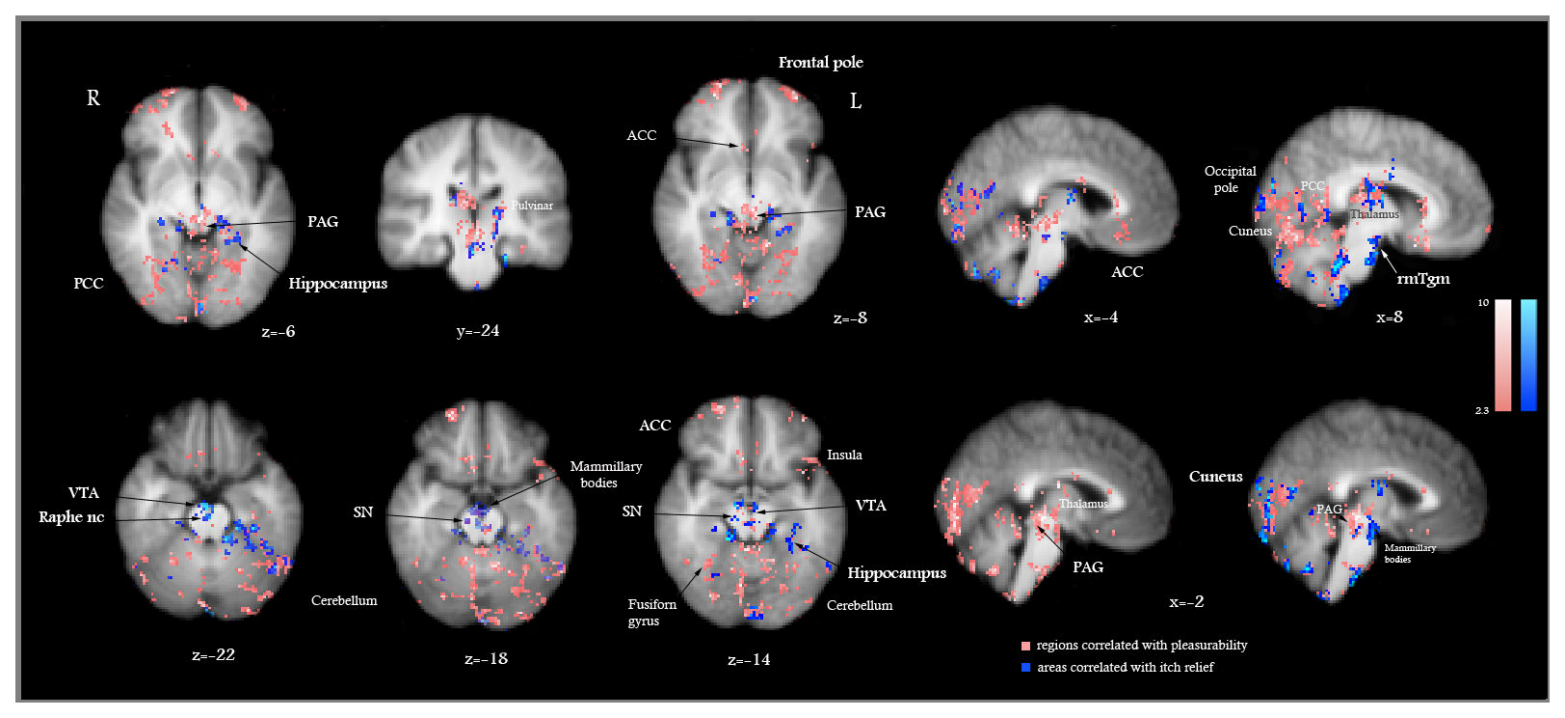
Brain areas significantly correlated with the pleasurability of scratching (pink) and with itch relief (blue) induced by actively scratching an itch, as identified by multiple regression analysis. VAS ratings of pleasurability and itch relief (calculated as the difference in itch ratings before and after scratching) were used as covariates of interest. PAG = periaqueductal gray; VTA = ventral tegmental area; SN= substantia nigra; ACC = anterior cingulate cortex; PCC = posterior cingulate cortex; rmTgm = rostromedial tegmentum. Talairach standard space coordinates (x, y, z). Areas significantly correlated with a Z score > 2.3, p < 0.05 are displayed. The color tones displayed correspond to Z score values as depicted in the color bars (at the same scale). R = Right; L = Left.

**Figure 6:**
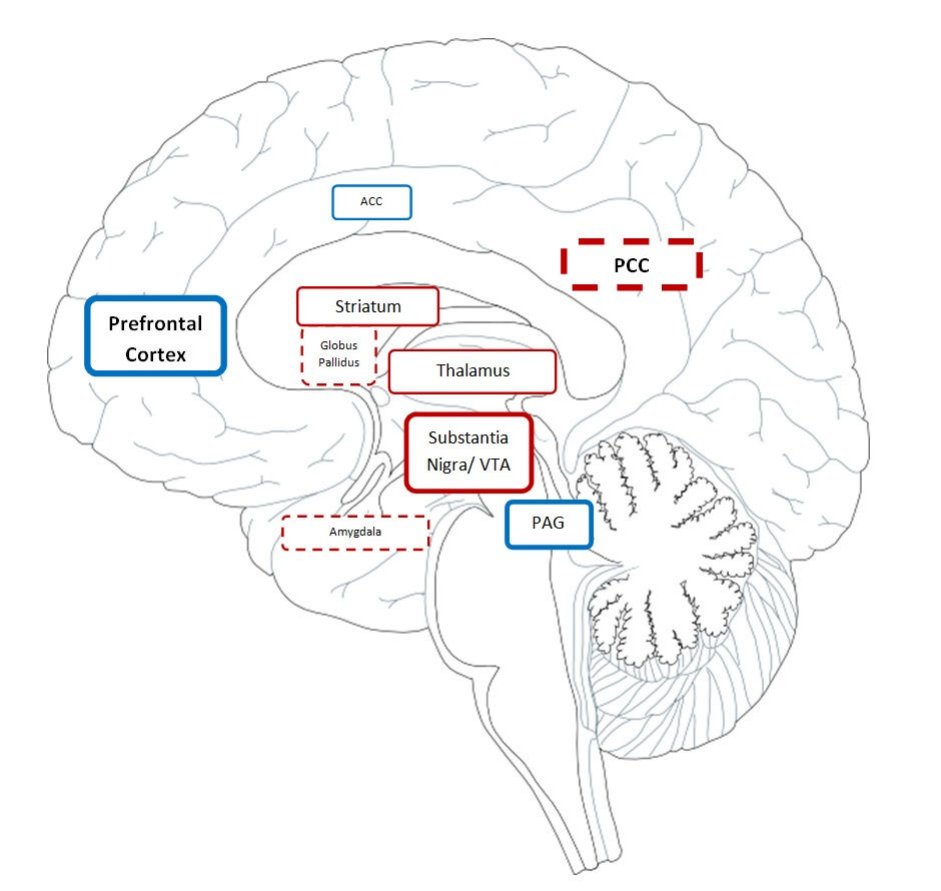
The major brain areas associated with the pleasurability of active scratching as revealed by GLM regression analysis. Regions which were activated (red) or deactivated (blue) by scratching are depicted. __(solid line) = positive correlation; ---- (dashed line) = inverse correlation with pleasurability ratings. Box size for each area is proportional to the magnitude of Z scores obtained in the regression analysis.

Regression analyses of brain activity versus pleasurability ratings showed that areas deactivated while actively scratching an itch coincided with areas significantly correlated with pleasurability. These areas are presented in Table 1 and Figures 5-6. Multiple thalamic nuclei, discrete areas in the midbrain VTA, PAG, substantia nigra (SN), hippocampus, amygdala, mammillary bodies and ACC, along with the ventral medial prefrontal cortex (vmPFC), were among the most significant regions associated with pleasurability.

**Table 1:** Brain areas correlated with the pleasurability of active scratching an itch, identified by regression analysis.

***Brainarea***	*x*	*y*	*z*	Z score
*Midbrain*				
VTA	-2	-16	-8	2.91
	2	-16	-14	2.39
	-2	-24	-14	3.96
VTA (rmTgm)	2	-24	-20	5.58
Raphé nucleus	4	-26	-8	4.33
	-2	-26	-8	6.08
Red Nucleus	6-8	-20	-8	3.04
	4	-22	-8	3.16
	-4	-24	-8	2.75
PAG	4	-34	-8	3.00
	-2	-30	-8	2.95
	-4	-38	-8	3.86
	-2	-32	-8	4.31
	-4	-30	-8	5.52
	-2	-30	-6	3.71
	0	-28	-6	7.44
	2	-28	-6	2.72
Sb. nigra	-14	-22	-8	2.51
	-14	-24	-8	5.80
*Thalamus*				
VPM	-14	-22	0	2.86
	-16	-20	2	2.50
VPL	-18	-22	0	2.37
VL	12	-12	0	3.45
Mediodorsal nc.	12	-20	8	2.64
	-6	-12	8	2.33
	-8	-12	8	2.41
	-8	-16	8	5.67
Ventral Anterior nc.	14	-6	10	2.63
VPM	-12	-18	6	2.49
Pulvinar	-16	-24	8	6.40
	-20	-26	8	5.58
	-14	-26	8	4.63
	-20	-24	8	3.46
Mammillary body	14	-18	0	3.49
	6	-12	-14	3.07
	14	-16	0	2.82
	12	-20	0	3.93
Hippocampus (cornu Ammonis)	-22	-42	2	4.61
	-22	-40	2	5.61
Parahippocampal gyrus	-24	-42	-6	5.03
ACC	-4	32	8	2.96
ACC	2	18	-8	3.71
	-4	30	-8	2.66
PCC (BA 30)	4	-48	18	4.30
PCC (BA 23)	4	-56	18	5.31
PCC (BA 29)	4	-46	18	3.42
PCC (BA 31)	10	-68	18	3.48
S2	36	-6	18	2.91
Putamen	22	0	10	3.58
	30	-6	10	2.84
Precuneus	14	-66	22	2.72
	20	-62	22	2.33
	16	-64	22	2.56
Cuneus (BA 18)	8	-70	22	2.33
Cuneus	6	-80	42	3.12
Lingual gyrus	-26	-64	2	3.54
Fusiform gyrus	-14	-74	2	3.17
Occipital pole	8	-88	22	6.21
	12	-88	22	3.29
Sup. frontal gyrus	22	64	-8	5.06
Middle frontal gyrus	-34	58	-8	6.50
Inf. frontal gyrus	-48	20	-8	2.52

Talairach standard space coordinates (x, y, z). Z score > 2.3; p< 0.05. BA = Brodmann area; ACC = anterior cingulate cortex; PCC = posterior cingulate cortex; VTA = ventral tegmental area; PAG = periaqueductal gray; VPL= ventral posterolateral nucleus; VPM = ventral posteromedial nucleus; S2 = secondary somatosensory area; VL = ventrolateral nucleus; rmTgm = rostromedial tegmentum.

### Brain areas associated with itch relief

We have also performed a regression analysis of brain areas whose activity correlated with the decrease in the ratings of itch perception, during actively and passively scratching an itch (Table 2). In contrast to pleasurability, relatively fewer areas were significantly correlated with itch relief: discrete zones in the cerebellum, globus pallidus and the mediodorsal nucleus of the thalamus, bilaterally (Table 2). The partial overlap between brain areas correlated with itch relief and the areas correlated with pleasurability is presented in Figure 5. 

**Table 2:** The brain areas significantly correlated with itch relief during the processing of active scratching.

***Areas correlated with itch relief***	*x*	*y*	*z*	Z score
Thalamus	-8	-8	14	2.46
Pulvinar	-14	-24	14	3.03
	-4	-8	12	2.87
Ventral lateral nucleus	-8	-10	6	2.46
	4	-14	12	2.99
	-4	-10	-14	3.23
Mediodorsal nc.	6	-14	12	3.50
	10	-18	12	2.89
	-4	-10	12	2.79
Cuneus	14	-78	14	3.87
Lingual gyrus	4	-80	12	4.21
Putamen	26	-2	12	2.40
Caudate body	14	-2	12	2.78
PCC	-4	-68	14	2.33

The ratings of itch relief were calculated as the difference between initial itch intensity and actual itch after scratching was performed. Talairach standard space coordinates (x, y, z). Z scores > 2.3, p < 0.05. PCC = posterior cingulate cortex.

## Discussion

One of the most intriguing neurobiological questions related to scratching an itch is why scratching is so pleasurable and what role it plays in relieving itch perception. In this study, we have discovered that brain responses evoked by active scratching implicate multiple structures previously described to process reward: VTA, NAc, the caudate nucleus and the ventromedial prefrontal cortex (vmPFC). The evoked responses in most of these brain areas were correlated with the pleasurability of scratching, while other areas were associated with itch relief. There was a limited degree of overlap between these regions, which suggests that perceiving itch relief and pleasurability are not identical processes. One of the most interesting results is that most areas correlated with pleasurability were virtually identical with areas of deactivation evoked by actively scratching an itch (Table 1).

The **midbrain** emerges as an important structure involved in the processing of scratching and in encoding the reward perceived in the active scratching of itch. The VTA, PAG, substantia nigra (SN) and the raphé nucleus appear to play a major role in the rewarding aspect of scratching, since all of these areas where strongly correlated with pleasurability ratings. VTA and the raphé nucleus were deactivated by actively scratching in comparison with itching alone, which suggested that the craving and frustration elicited by itch were successfully quenched by active scratching. Furthermore, passive scratching was not able to elicit deactivation of these areas. In particular, the PAG was found to be significantly deactivated by active scratching in rapport with the itch state. This raises the possibility that self-scratching leads to a marked inhibition of PAG. It is well-known that activation of reward structures via prefrontal and limbic system connections leads to effects on PAG activity. These relays are important in the modulation of pain via the descending inhibitory control mechanisms [6]. Our results suggest that an opposite effect operates in itch modulation during active scratching. Since nociceptive interneurons at the spinal level inhibit itch transmission, it is suggested that itch can be modulated by the deactivation of PAG and of the raphé nucleus. 

A previous study in non-human primates investigating the mechanism of itch inhibition by scratching showed that the effect on itch-specific spinal neurons was state-dependent, being active only in the presence of itch [7]. The state-specific inhibition could be regulated by modulatory descending inputs from above [8]. We surmise this top-bottom modulation could be mediated by supraspinal circuits involved in craving, reward or motivation. The output from the reward circuit leads to the deactivation of raphé nucleus and PAG during active scratching, which could represent a principal mechanism for itch inhibition. This is a unique feature that appears to distinguish the modulation of itch from pain. 

Multiple anatomic subdivisions of the ventral and dorsal striatum: the nucleus accumbens, caudate, putamen, previously attributed roles in encoding motivation, expectation or pleasure, were associated with the pleasurability of scratching, a finding which is in good agreement with other studies: the putamen was linked with the relief of itch induced by acupuncture [9], and with passively scratching an itch contrasted to scratching alone [4]. 

A possible connection at the level of midbrain between the activation of motor relays coordinating the voluntary motor response - inherent in scratching motions - and the activation of reward circuits is strongly suggested. The activity observed in the red nucleus and substantia nigra (as well as in the cerebellum) is not surprising, as these areas are classically involved in motor coordination; however, their activation was also strongly correlated with pleasurability. Noteworthy, substantia nigra is a major source of dopamine in the nigrostriatal and mesolimbic circuits. Our results suggest that the association of motor-related activity with pleasurability may have a basis in the implication of midbrain structures. Substantia nigra and VTA could represent the key areas encoding the pleasurability of scratching. Behavioral and pharmacological studies of dopamine pathways have led to the association of the mesolimbic pathway with reward processing and of the nigrostriatal pathway with motor activity; recently, both circuits have been associated with reward [10]. 

It is worthy of mention that besides the involvement of midbrain structures, active scratching, which provided a higher pleasurability and itch relief, induced a more extensive deactivation of the anterior cingulate cortex, prefrontal cortex, insula and the lentiform nucleus, other main areas involved in processing reward. This effect was much stronger in comparison with passive scratching. 

In addition, our results suggest that thalamus has a significant role in processing the variable sensory information of perceiving itch while scratching. Multiple nuclei in the thalamus were significantly activated by active scratching of itch, and were correlated with pleasurability and itch relief. The activity of VPL, a known station processing itch information projecting to the cortex, was correlated with itch relief. The anterior nucleus of thalamus could play a role in the assessment of itch reduction, due to its activation during active scratching. The pulvinar was significantly activated during self-scratching and its response was correlated with the perceived pleasurability. The medial dorsal nucleus (MDNc), well-known to be involved in reward circuits and top-bottom mechanisms via reciprocal connections to the orbitoprefrontal cortex [11], was correlated both with pleasurability and itch relief. In light of these observations, we conclude that the MDNc coordinates sensory and motor inputs with the reward circuit activity during scratching via connections with ACC, PFC and PAG [12]. The MDNc is also the major relay for working memory and provides context for cognitive-motivational processes [13]. 

Active self-scratching recruited stations of the reward circuit: NAc, VTA, vmPFC, SN, emphasizing the addictive or reinforcing character of this behavior. In contrast, only parts of these circuits were stimulated by passive scratching: ACC and vmPFC. Passive scratching uniquely activated the subthalamic nucleus, a formation that has also been implicated in reward [5]. Our results suggest that dopaminergic pathways are implicated in the rewarding, addictive phenomenology of the itch-scratch cycle. The reward-system activity associated with itch relief overlapped only to a limited extent with the areas correlated with the pleasurability of scratching. In this respect, our results slightly differ from studies of pain, which suggest that the same circuits process reward and analgesia [14,15]. 

In conclusion, the engagement of reward circuits during active scratching may represent a major route by which antipruritic top-down mechanisms are initiated. Our data indicate that the reward is dually perceived as a decrease in itch intensity and a pleasurable input that accompanies scratching. These co-existing, synergistic mechanisms combine to counter the negative emotional-affective impact of itch.

## Methods

### Subjects

14 healthy volunteers: 8 females, 6 males, with ages 19-58 (average age 30.3 ± 9.2) were enrolled. Subjects were free of any skin disease and were not using any systemic or topical medications. A written informed consent was obtained. The protocol was approved by the Internal Review Board of Wake Forest University Health Sciences. The study was conducted in agreement to the principles of Declaration of Helsinki. 

### Design and sequence of the scratching operations

Two baseline scans at rest were acquired first. One fMRI scan was acquired separately for passive or active scratching intervention without itch and was used as a distinct control. For the passive scratching series, the participants were exogenously scratched by an investigator (see below). The itch condition alone - without scratching - was imaged separately. Acute experimental itch was induced by applying cowhage spicules on the distal volar side of the right forearm, as previously described [16,17]. For the fMRI series that investigated the effects of scratching an itch, itch was re-induced with cowhage in a different area of the volar side of the right forearm. Immediately after itch induction (60 seconds post-application) spicules were carefully removed with adhesive tape (Scotch, 3M, USA) and the itchy area was scratched, either passively by an investigator, or actively by subjects themselves (in two separate scans, respectively). Passive and active scratching sessions were randomized to minimize potential order effects.

Active scratching was performed continuously by study participants themselves during the entire scan period (4 min 54 s) using only the tips of the fingers of the left hand to scratch a distal site on the right forearm (2-3 cm above the wrist), while strictly refraining from other movements and minimizing larger movements of the left arm. To easily reach the right forearm (with the left hand), the right hand was placed on the chest in the most comfortable position possible while lying (supine) inside the scanner.

Passive scratching was delivered by a study investigator using a MediPak cytology brush no. 7 (General Medical Corporation, Elkridge, MD) which applied a constant force equivalent to a weight of 29 g, as described previously [2,3].

### Statistical analysis of psychophysical data on itch intensity, scratching pleasurability and itch relief

A mixed model analysis accounting for subject as a random factor and adjusted for itch while scratching was performed to compare pleasurability between the active and passive itch while scratching, using PASW 18.0 software (SAS; Chicago, IL). Paired t tests were performed to compare pleasurability for each form of scratching vs. its own control (scratching without itch), and the decrease in itch ratings by active or passive itch with scratching to its itch control. Mixed model analysis with subject as a random factor was used to compare itch relief (as the difference in itch ratings between itch while scratch vs. itch alone) between active vs. passive modalities.

### Functional MRI

All experiments were carried out on a GE 1.5T TwinSpeed scanner (GE Healthcare, Milwaukee, WI) with an eight-channel phased array receive-only head coil (Invivo Devices, Gainesville, FL) for data collection. PseudoContinuous Arterial Spin Labeling consisted of series of 800 µs, 25 degree Hanning window-shaped RF pulses. Repetition time between pulses was 1736 µs; gradient residual moment was set at 13.6%. Labeling duration was 1600 ms with a post-labeling delay of 1000 ms. Control and label imaging pairs were acquired by changing the phase of alternating RF pulse by 0 degrees and 180 degrees, respectively. Two non-selective inversion pulses were interleaved among the pCASL RF pulses and the post-labeling delay at 673 ms and 2219 ms. *Propeller acquisition*. For 3D-GRASE-Propeller, a number of rectangular volumes (known as bricks) were acquired at different rotation angles relative to the central k_z_-axis. Each brick was sampled with a multi-shot 3D-GRASE readout module. Eight bricks were evenly distributed from 0 to 157.5° by an incremental angle of 22.5°. The number of bricks acquired for each image satisfied the Nyquist sampling criterion and ensured adequate perfusion Signal-to-Noise-Ratio. Further reduction in through-plane blurring was achieved by employing a partial-Fourier multi-shot [18]. The partial-Fourier multi-shot encoding along the slice encode direction resulted in full brain coverage (31 slices) with 5 mm slice thickness using 24 encodings. Each brick was acquired with a positive and a negative frequency-encoding gradient during the EPI readout. The toggling of the frequency-encoding gradient allowed Nyquist ghosts to be removed using the self-referenced correction method GESTE. The acquisition pattern for each Propeller brick was Control/Label ASL pairs in the innermost loop, multi-shot slice encoding, then frequency encoding gradient alteration in the outermost loop. The angle of the next Propeller brick was increased by 22.5 degrees and the cycle was repeated. The total scan time was 4 minutes and 54 seconds [*T*_*scan time*_ = *T*_*R*_ × *(N*_*ASL*_ × *N*_*Blade*_ × *N*_*Shots*_ × *N*_*GESTE*_ + 2)], where TR was the repetition time (3000 ms). The total scan time included 2 additional scans to achieve steady state. Rotational and translational motion correction was performed along the in-plane direction. The same motion correction reference image was used for both control and label blades during the in-plane motion correction. The corrected blades were gridded onto a Cartesian coordinate space with uniform-density compensation. The final image for each slice was obtained by performing a 2D Fourier transform on *k*-space data. The control and label images were reconstructed separately and then subtracted to obtain the perfusion weighted image. High resolution anatomical images were acquired using 3D T_1_ weighted SPGR for tissue segmentation. Imaging reconstruction software was written in Matlab (Mathworks, Natick, MA) using functions from the National Center for Image Guided Therapy Fast Imaging Library.

### Data analysis

The functional image analysis package FSL (Functional Magnetic Resonance Imaging of the Brain Software Library, Center for FMRIB, University of Oxford, Oxford, UK) was used for image processing and statistical analysis. The Cerebral Blood Flow (CBF) data was movement corrected and spatially smoothed using a 5 mm 3D isotropic Gaussian kernel. Each CBF image was scaled by its mean global intensity (intensity normalization) to minimize variability due to global CBF changes. Next, each subject's CBF images were registered to their structural data using a seven-parameter linear 3D transformation and transformed into standard stereotaxic space as defined by the Montreal Neurological Institute (MNI), using a 12-parameter linear 3D transformation. Standard general linear model-based analyses using fixed-effects models within subjects and random effects models (FLAME1+2) between subjects were performed to analyze the contrasts between the series of interest versus their corresponding control conditions, by paired t test as follows: 1) scratching an itch, either actively or passively, was compared to itch condition itself. 2) Scratching an itch was contrasted with scratching in the absence of itch, for either active or passive scratching. 3) Higher level analysis was performed to compare the effects of active scratching with passive scratching, either in the presence or absence of itch. The average effect for each condition was also studied for the entire group. To identify the areas correlated with itch relief and pleasurability, a multiple regression analysis using the VAS ratings as covariates of interest, respectively, was performed under the General Linear Model (GLM), setting up individual orthogonalization of ratings versus main effect in order to prevent overestimation. All analyses were performed as whole-brain analyses. Statistical significance for whole brain analysis was set at p<0.05, Z score > 2.3. 

## Supporting Information

Table S1 **Regions of interest involved in the brain processing of itch significantly impacted by scratching.** Brain areas correlated with itch relief and scratching pleasurability are shown. The highest Z scores > 2.3 are presented for most significantly correlated clusters. *Deactivated in a higher-level contrast analysis in comparison to itch condition. ^***#***^ Results of regression analyses performed for active scratching an itch are displayed. BA = Brodmann area; ACC = anterior cingulate cortex; PCC = posterior cingulate cortex; VTA = ventral tegmental area; PAG = periaqueductal gray; VPL= ventral posterolateral nucleus; VPM = ventral posteromedial nucleus; AN = anterior nucleus; LON = lateral posterior nucleus; LDN = lateral dorsal nucleus; MD = mediodorsal nucleus.
(DOCX)
