# Correction: Brain’s Reward Circuits Mediate Itch Relief. A Functional MRI Study of Active Scratching

**DOI:** 10.1371/annotation/c58aebe3-8f01-4c14-991a-c229e35b8f74

**Published:** 2014-01-24

**Authors:** Alexandru D. P. Papoiu, Leigh A. Nattkemper, Kristen M. Sanders, Robert A. Kraft, Yiong-Huak Chan, Robert C. Coghill, Gil Yosipovitch

This article was republished on January 24, 2014 to include correct figures in both the online and PDF versions of the article. 

