# Supplementary material for: Brain’s Reward Circuits Mediate Itch Relief. A Functional MRI Study of Active Scratching
Source: PLoS One. 2013 Dec 6;8(12):e82389. doi: 10.1371/journal.pone.0082389 (PMC3855767; doi:10.1371/journal.pone.0082389)
Supplement: Table S1 — Regions of interest involved in the brain processing of itch significantly impacted by scratching. Brain areas correlated with itch relief and scratching pleasurability are shown. The highest Z scores > 2.3 are presented for most significantly correlated clusters. *Deactivated in a higher-level contrast analysis in comparison to itch condition. # Results of regression analyses performed for active scratching an itch are displayed. BA = Brodmann area; ACC = anterior cingulate cortex; PCC = posterior cingulate cortex; VTA = ventral tegmental area; PAG = periaqueductal gray; VPL= ventral posterolateral nucleus; VPM = ventral posteromedial nucleus; AN = anterior nucleus; LON = lateral posterior nucleus; LDN = lateral dorsal nucleus; MD = mediodorsal nucleus. (DOCX) [file pone.0082389.s001.docx]

**Supplemental Table S1**. Regions of interest involved in the brain processing of itch significantly impacted by scratching.

| *Brain area* | Itch | Actively  scratching an Itch | Passively scratching an Itch | Correlation with pleasurability of scratching^#^ | Correlation with itch relief induced by scratching^#^ |
| --- | --- | --- | --- | --- | --- |
| Primary somatosensory | Activated | Activated | Activated | Inverse  BA3; Z=4.8 | Inverse  BA1,2,3 Z=4.4 |
| Secondary somatosensory | Activated | Activated | Activated | Inverse  BA40; Z=2.3 | Inverse  BA40; Z=3.2 |
| Primary motor | Activated | Activated | Deactivated | Inverse  BA4; Z=5.7 | Inverse  BA4; Z=5.4 |
| Supplementary motor area | Activated | Activated |  | Inverse  BA5,6,7  Z=4.3 | Inverse  BA5,6,7 Z=5.5 |
| **Prefrontal cortex** |  |  |  |  |  |
| Dorsolateral | Activated | Activated |  | Positive BA9,46; Z=10 Inverse  BA8 Z=6.9 | Inverse  BA8;  Z=4.8 |
| Frontal pole | Activated | Deactivated | Deactivated | Positive  extensive BA10; Z=10.0 |  |
| Orbitofrontal | Activated | Deactivated | Deactivated | Positive  BA11; Z=8.1 |  |
| Ventrolateral | Activated | Deactivated | Deactivated | Positive  BA45; Z=3.2 |  |
| ACC | Activated | Activated or deactivated in different regions | Deactivated | Positive  BA32,33 Z=5.7 | Positive  BA24;  Z=3.5 |
| PCC | Activated | Activated | Activated | Inverse  (extensive) BA23, 31 Z=5.4 | Inverse  (extensive) BA23, 31 Z=9.7 |
| Precuneus | Activated | Activated | Activated | Positive  BA31; Z=8.3  Inverse  BA7; Z=9.3 | Positive  BA7; Z=4.2  Inverse  BA31; Z=8.8 |
| Parahippocampus | Activated | Deactivated | Activated | Positive  Z=10.8 | Positive  Z=9.0 |
| Hippocampus | Activated | Deactivated | Activated | Positive  Z=4.7 | Inverse Z=5.2 |
| Amygdala | Activated | Deactivated |  | Inverse Z=5.4 | Inverse Z=4.6 |
| Insula | Activated | Deactivated | Deactivated |  |  |
| **Thalamus** | Activated | Activated | Deactivated |  |  |
| VPL |  |  |  | Positive Z=2.8 |  |
| VPM |  |  |  | Positive Z=2.7 | Positive Z=2.6 |
| LPN |  |  |  | Positive Z=7.2 | Positive Z=3.6 |
| LDN |  |  |  | Positive Z=7.9 | Positive Z=3.5 |
| AN |  |  |  | Positive Z=6.8 | Positive Z=6.3 |
| MD |  |  |  | Positive Z=7.4 | Positive Z=7.7 |
| Pulvinar |  |  |  | Positive Z=4.6 | Positive Z=2.7 |
| **Striatum** |  |  |  |  |  |
| Caudate |  | Activated |  | Positive  (caudate head) Z=7.9 | Positive  (caudate body) Z=4.2 |
| Putamen | Activated |  | Deactivated | Inverse  Z=4.3 | Positive Z=4.9 |
| Nc. accumbens |  | Deactivated* |  |  |  |
| Globus pallidus | Activated |  |  | Positive  Z=6.0 | Positive  Z=4.5 |
| Substantia nigra |  |  |  | Positive  Z=4.4 | Positive  Z=4.8 |
| Subthalamic nucleus |  |  | Activated |  |  |
| **Midbrain** |  |  |  |  |  |
| VTA |  | Deactivated* |  | Positive  Z=4.7 | Positive  Z=6.0 |
| Red nucleus |  | Deactivated* |  |  |  |
| Dorsal nucleus of the raphé |  | Deactivated* |  | Positive  Z=4.1 | Positive  Z=6.5 |
| PAG |  | Deactivated* |  | Positive  Z=4.1 |  |

Notes. Brain areas correlated with itch relief and scratching pleasurability are shown. The highest Z scores > 2.3 are presented for most significantly correlated clusters.

BA = Brodmann area. ACC = anterior cingulate cortex; PCC = posterior cingulate cortex; VTA = ventral tegmental area; PAG = periaqueductal gray; VPL= ventroposterolateral nucleus; VPM = ventroposteromedial nucleus; AN=Anterior nucleus; LPN = lateral posterior nucleus; LDN = lateral dorsal nucleus; MD = Mediodorsal nucleus.

*Deactivated in a higher-level contrast analysis in comparison to itch condition.

^#^ Results for regression analyses performed for active scratching an itch are displayed.
